# Negative Pressure Promotes G3BP1-Mediated Migration of Corneal Epithelial Cells Through Activation of AKT/ERK/Paxillin Pathway

**DOI:** 10.3390/ijms27167273

**Published:** 2026-08-14

**Authors:** Chia-Hui Lai, Pang-Hung Hsu, Chih-Chin Hsu, Chien-Tzung Chen, Yu-Chiau Shyu, Jong-Hwei Su Pang, Chi-Chin Sun

**Affiliations:** 1 Graduate Institute of Clinical Medical Sciences, College of Medicine, Chang Gung University, Taoyuan 333, Taiwan; jaihuey@gmail.com; 2 Department of Bioscience and Biotechnology, National Taiwan Ocean University, Keelung 202, Taiwan; phsu@ntou.edu.tw (P.-H.H.); yuchiaushyu@gmail.com (Y.-C.S.); 3 Department of Physical Medicine and Rehabilitation, Chang Gung Memorial Hospital at Keelung, Keelung 204, Taiwan; steele0618@gmail.com; 4 Division of Plastic and Reconstructive Surgery, Chang Gung Memorial Hospital at Keelung, Keelung 204, Taiwan; ctchenap@cgmh.org.tw; 5 Department of Plastic and Reconstructive Surgery, Craniofacial Research Center, College of Medicine, Chang Gung University, Taoyuan 333, Taiwan; 6 Community Medicine Research Center, Chang Gung Memorial Hospital, Keelung Branch, Keelung 204, Taiwan; 7 Department of Ophthalmology, Chang Gung Memorial Hospital at Keelung, Keelung 204, Taiwan; 8 School of Medicine, College of Medicine, Chang Gung University, Taoyuan 333, Taiwan

**Keywords:** negative pressure, cell migration, G3BP1, AKT/ERK/paxillin signaling, corneal epithelial wound healing

## Abstract

The corneal epithelium serves as the outermost transparent barrier of the eye and depends on rapid and coordinated cellular responses for wound repair. Although negative pressure (NP) has been shown to accelerate wound healing in other tissues, its cellular and molecular effects on corneal epithelium remain undefined. This study investigates how NP regulates corneal epithelial cell physiology and identifies the molecular mechanisms underlying NP-induced migration. Human corneal epithelial cells were exposed to normal or NP conditions, and cell motility was quantified using scratch-wound and transwell migration assays. Nuclear and cytoplasmic fractions were isolated for proteomic profiling to identify NP-responsive proteins. G3BP1 was selected as a candidate regulator and subsequently examined using molecular, biochemical, and functional assays to determine its role in NP-mediated signaling. Proteomic analysis revealed a significant NP-induced upregulation of G3BP1. Mechanistically, G3BP1 suppressed epithelial junctional proteins, including E-cadherin, p120-catenin, and ZO-1, while activating key pro-migratory signaling pathways involving AKT, ERK1/2, FAK, and Paxillin. These coordinated changes enhanced cytoskeletal dynamics and promoted corneal epithelial cell migration under NP stimulation. G3BP1 functions as a critical mechanotransduction mediator of NP, orchestrating adhesion remodeling and activating pro-migratory signaling cascades to facilitate corneal epithelial cell motility. These findings reveal a previously unrecognized cellular mechanism through which NP promotes epithelial repair and highlight G3BP1 as a potential therapeutic target for persistent corneal epithelial defects.

## 1. Introduction

The cornea, together with the tear film, constitutes the outermost protective layer of the ocular surface [1], playing a critical role in maintaining visual clarity and shielding the eye from external insults. When this layer is damaged and wound healing is delayed, corneal transparency is compromised, leading to visual impairment [2]. Persistent corneal epithelial defect (PCED) is defined as the failure of the corneal wound healing process to complete within 14 days despite conventional management. PCED is associated with several clinically significant morbidities, including irregular corneal astigmatism [3], microbial keratitis [4], corneal opacities [5], stromal thinning [6], corneal melting, perforation, endophthalmitis and phthisis bulbi [7].

Among the various etiologies contributing to PCED, diabetic keratopathy is a major risk factor. Corneal wounds in diabetic patients often exhibit resistance to conventional treatments, potentially progressing to neurotrophic corneal ulcers, secondary infections, and vision loss [8]. The wound healing response following corneal epithelial damage is a complex and intricate process. It involves several steps, including cellular proliferation, differentiation, migration, apoptosis, and extracellular matrix remodeling to rebuild the integrity of the corneal epithelium [9]. These processes are tightly regulated by various growth factors and extracellular matrix proteins such as epidermal growth factor (EGF), keratinocyte growth factor (KGF), transforming growth factor (TGF)-β, platelet-derived growth factor (PDGF), insulin-like factor (IGF), inflammatory cytokines [10], and proteinases like matrix metalloproteinases (MMPs) [11]. If the damage is not properly repaired, it may affect the transparency and reflectivity of the cornea, leading to poor visual outcomes [8].

Current therapeutic options for PCED include artificial tears (ATs) [12], bandage contact lenses (BCLs) [13], topical epidermal growth factor (EGF) [14], autologous serum eye drops (ASEDs) [7], amniotic membrane transplantation (AMT), and corneal transplantation [15]. Recombinant human epidermal growth factor (rhEGF) eye drops promote centripetal migration of corneal limbal stem cells by activating the EGF receptor to accelerate corneal wound repair [16]. Similarly, ASED contains growth factors, fibronectin, and vitamins, supporting corneal epithelial proliferation, migration, and differentiation while exhibiting antibacterial properties [7]. However, while AMT serves as an effective biological dressing with anti-inflammatory, anti-fibrotic, and anti-angiogenesis properties, its semi-transparency may temporarily hinder visual acuity. Corneal transplantation carries risks of infection and rejection [17]. Therefore, there is an ongoing need for novel, effective therapeutic approaches to expedite corneal repair and safely restore patients’ vision.

Recently, Negative Pressure Wound Therapy (NPWT) has been proven to be effective in accelerating healing for chronic and refractory wounds, such as diabetic foot ulcers [18], pressure ulcers [19], and acute traumatic wounds [20]. This specific NPWT system consists of a porous foam dressing receiving continuous or intermittent suction from an electronically powered suction device, achieving a sub-atmospheric pressure that is 125 mmHg below ambient pressure [21]. NPWT enhances wound drainage, prevents secondary infection, and maintains a moist environment conducive to cell proliferation. Additionally, a negative pressure environment may influence the activity of fibroblasts, chemotactic signals, and proliferation within the fibrous protein clot matrix [22]. It is hypothesized that negative pressure may destabilize cell adhesion proteins such as ZO-1 and E-cadherin, thereby promoting cell migration and facilitating wound healing [23]. However, most NPWT studies focus on its effect on cell proliferation and fibroblast activity, with limited exploration of its impact on epithelial cell migration. Moreover, the concept of NPWT has never been applied to corneal wound healing, and its effects on corneal epithelial cells remain unknown.

G3BP1 is known to regulate the cell migration of various cancer cells [24,25,26] by interacting with Ras-GAP (GTPase-activating protein) [27]. Previous research has shown that G3BP1 is associated with signaling pathways involved in tumorigenesis and metastasis, such as Ras signaling [27] and Wnt/β-catenin signaling [24]. Although G3BP1’s role in regulating cell migration is well-documented in cancer cells, its functional role in corneal epithelial cells, particularly the influence of NP-increased G3BP1 expression on cell migration, has not yet been studied.

Thus, the purpose of this study is to investigate the effect of NPWT on corneal epithelial cell proliferation and migration and explore the underlying molecular mechanisms, focusing on the role of G3BP1 in regulating cell migration. Understanding these processes could lead to the development of novel therapeutic approaches to accelerate corneal wound healing and address the unmet clinical need for effective treatments for PCED.

## 2. Results

### 2.1. Effect of Negative Pressure (NP) on Viability and Migration of Corneal Epithelial Cells

**Figure 1:**
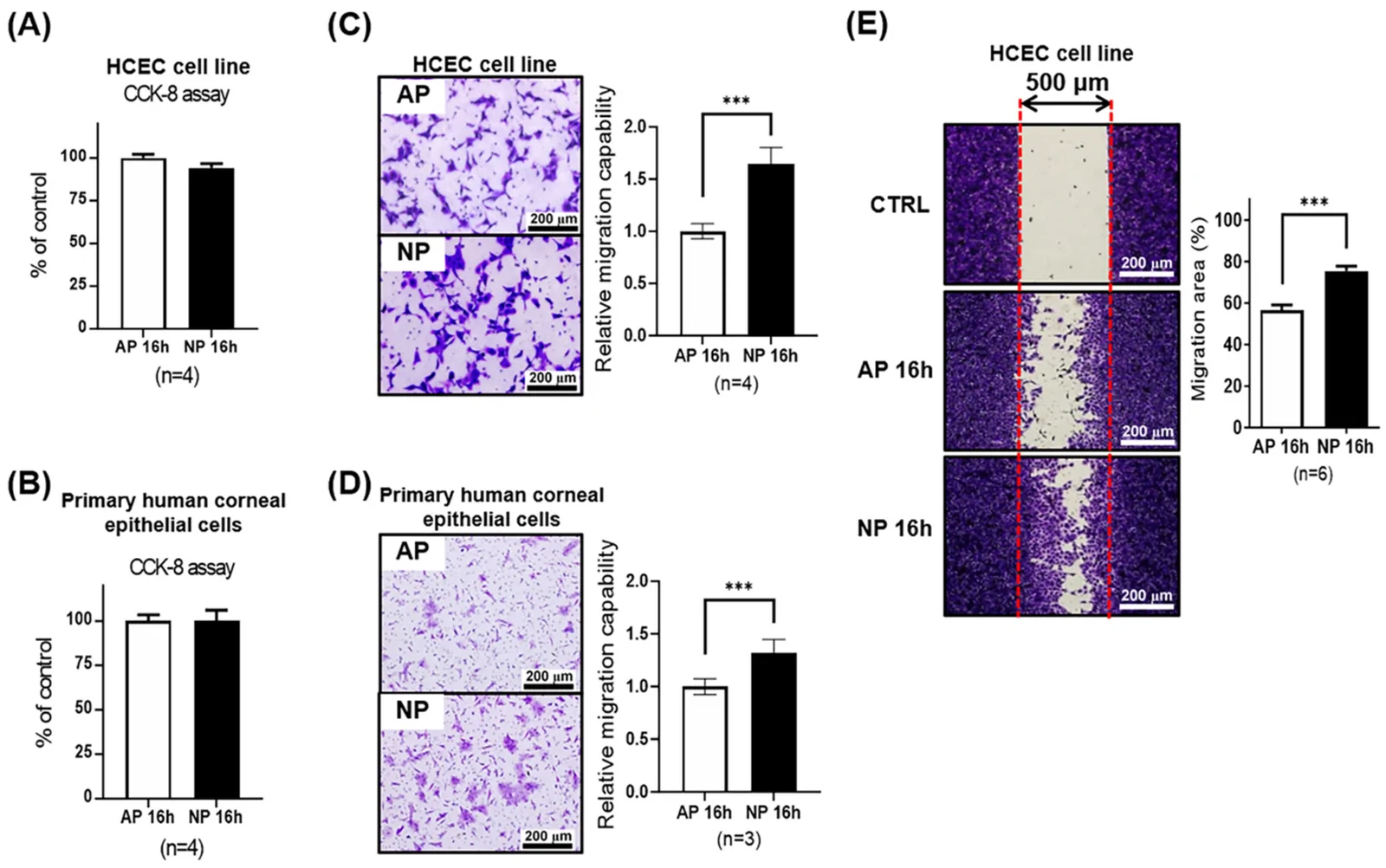
Effects of NP on proliferation and migration of corneal epithelial cells. (**A**,**B**) CCK-8 assay results showing the effects of atmospheric pressure (AP) and NP on cell viability in the HCEC cell line (**A**) and primary human corneal epithelial cells (**B**). Cell viability was measured by optical density (OD) at 450 nm. (**C**,**D**) Transwell migration assay showing increased migration of the HCEC cell line (**C**) and primary human corneal epithelial cells (**D**) following 16-h AP or NP treatment. Migrated cells were quantified and analyzed. (**E**) Wound healing assay demonstrating the migration of the HCEC cell line following 16-h AP or NP treatment. Migratory area was quantified using ImageJ. Representative images were stained with crystal violet (original magnification ×150; scale bar, 200 μm). Quantitative data are presented as mean ± SEM. *** *p* < 0.001 compared with control (CTRL). The red dashed lines indicate the boundaries of the initial cell-free gap.

To evaluate the effects of NP on corneal epithelial cells, cell viability of the HCEC cell line and primary human corneal epithelial cells was assessed under atmospheric-pressure (AP) and NP conditions for 16 h using the CCK-8 assay. NP did not significantly affect the viability of either the HCEC cell line (Figure 1A) or primary human corneal epithelial cells (Figure 1B).

The effects of NP on cell migration were further evaluated using a Transwell migration assay under the same conditions. NP treatment increased the migratory ability of the HCEC cell line by 1.65-fold (Figure 1C) and of primary human corneal epithelial cells by 1.36-fold (Figure 1D), compared with AP-treated controls.

To further assess the effects of NP on cell migration, a wound healing assay was performed. After 16 h of NP treatment, the migration rate of the HCEC cell line was significantly increased, with approximately 75% closure of the wound area compared with 56% under AP conditions (Figure 1E).

### 2.2. NP Reduces Membrane Protein Expression in Corneal Epithelial Cells

To further investigate the mechanism underlying NP-enhanced cell migration, we analyzed the protein distribution of the HCEC cell line under AP and NP conditions. Subcellular fractions, including cell membrane, cytosol, soluble nuclear, and chromatin-bound nuclear components, were isolated using a Subcellular Protein Fractionation Kit. The goal of this fractionation was to investigate the redistribution of membrane-associated proteins during the early stages of cell migration. Specifically, we aimed to determine whether NP conditions induced the loss or internalization of key membrane proteins associated with contact inhibition, which are essential for maintaining cell–cell adhesion. Protein concentrations in each fraction were quantified using a BCA assay, allowing calculation of the total protein content for each fraction and its proportion relative to total cellular protein. This analysis provided insights into the role of adhesion-associated proteins in facilitating cell migration under NP conditions.

**Figure 2:**
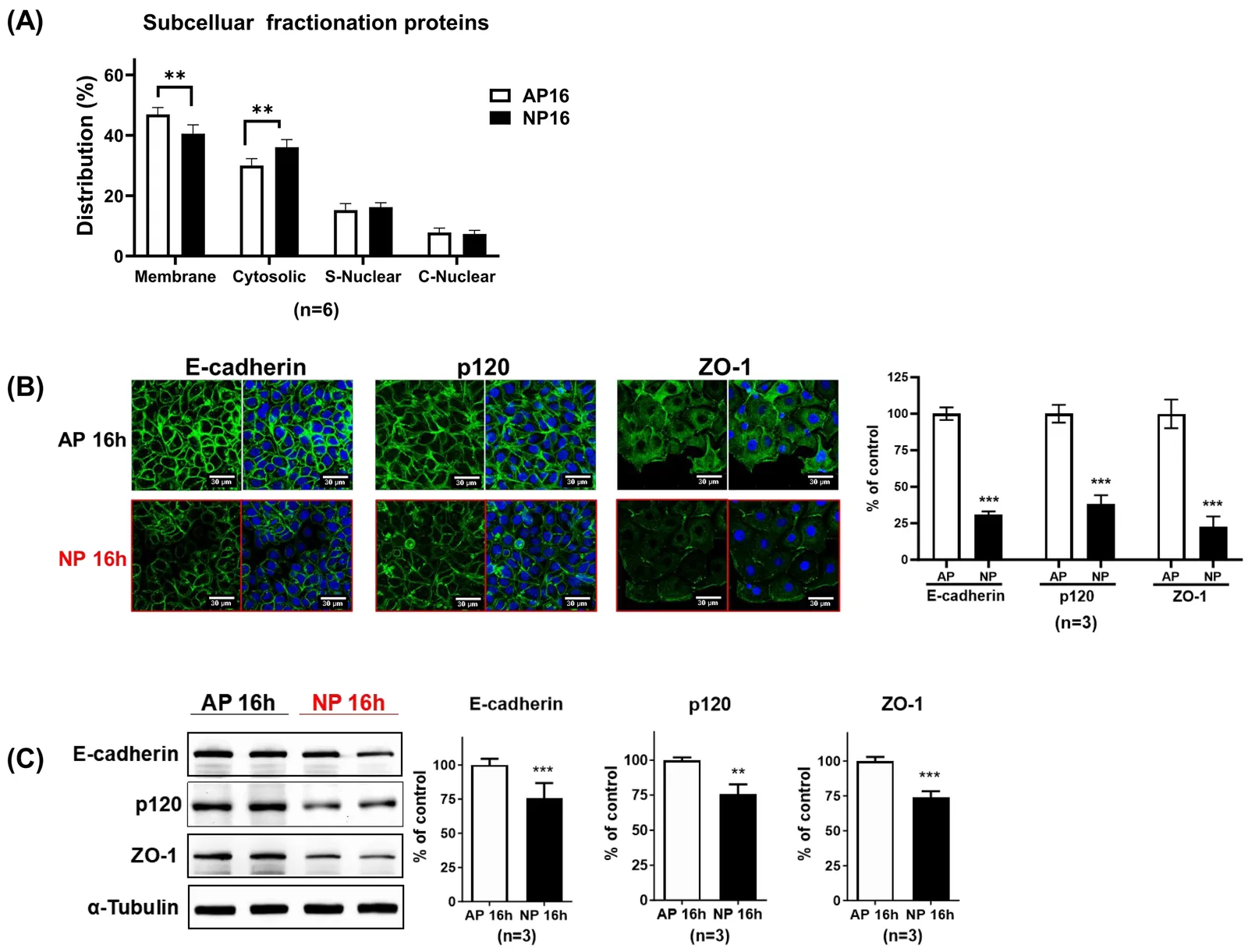
Differential expression of subcellular proteins in HCEC cell line under AP and NP conditions. (**A**) Subcellular fractionation of HCEC cell line following 16-h AP and NP treatments, showing protein distribution in membrane, cytoplasmic, and nuclear fractions (*n* = 6). Soluble nuclear (S-nuclear) and chromatin-bound nuclear (C-nuclear) fractions are indicated. (**B**) Immunofluorescence staining of E-cadherin, p120, and ZO-1 (green) at the cell membrane of the HCEC cell line after 16-h AP or NP treatment (upper panel). Nuclei were stained with DAPI (blue). Images were acquired at ×1000 magnification (scale bar, 30 μm). The bar graph shows the quantification of fluorescence intensity, expressed as a percentage of the AP control group. Data are presented as mean ± SEM, *** *p* < 0.001 vs. AP. (**C**) Western blot analysis of E-cadherin, p120, and ZO-1 expression in the HCEC cell line following 16-h NP treatment (lower panel, left; *n* = 3). Quantitative analysis of membrane-associated proteins, including E-cadherin (135 kDa), p120 (110 kDa), and ZO-1 (225 kDa), under AP and NP conditions (lower panel, right). Data are expressed as mean ± SEM. *** *p* < 0.001; ** *p* < 0.01.

The results showed that under AP conditions, the average protein distribution across cell membrane, cytosol, soluble nuclear, and chromatin-bound fractions was 47%, 30%, 17% and 6% of total cellular protein, respectively. However, under NP conditions for 16 h, protein content shifted to 41% (** *p* < 0.01) for the cell membrane and 36% (** *p* < 0.01) for the cytosol, while the protein contents of the soluble nuclear and chromatin-bound fractions remained unchanged. (Figure 2A). This significant shift in protein distribution suggests a potential mechanism whereby NP reduces the availability of membrane proteins, which may weaken cell–cell adhesion and promote cell migration.

To further investigate this hypothesis, we examined the expression of membrane proteins implicated in cell migration regulation, including E-cadherin, p120, and ZO-1, using immunofluorescent staining and Western blot analysis. Immunofluorescent staining demonstrated a marked reduction in the membrane localization of E-cadherin, p120, and ZO-1 in HCEC cell line after 16 h of NP treatment. Quantitative analysis of fluorescence intensity confirmed that the expression of E-cadherin, p120, and ZO-1 in the NP conditions was significantly lower than in the AP conditions, suggesting that negative pressure may impair cell–cell adhesion in HCEC cell line (Figure 2B). Western blot analysis further confirmed these findings, revealing reductions of 24.3%, 24.1%, and 25.9% in E-cadherin, p120, and ZO-1 expression, respectively, under NP conditions compared to AP conditions (Figure 2C). Similarly, in primary human corneal epithelial cells subjected to NP conditions, the expression levels of E-cadherin, p120, and ZO-1 decreased by 36.9%, 27.2%, and 30%, respectively, compared to cells under AP conditions. Fluorescence quantification showed a significant reduction in E-cadherin, p120, and ZO-1 in NP-treated cells compared to the AP conditions (Figure S2). These findings suggest that NP disrupts cell junction integrity in corneal epithelial cells.

### 2.3. NP Activates AKT, ERK1/2, FAK, and Paxillin Signaling in Corneal Epithelial Cells

To investigate the downstream signaling pathways activated by NP, we conducted proteomic analysis. The results identified that NP activates several migration-related signaling pathways, including AKT (protein kinase B), ERK1/2 MAP kinases (extracellular signal-regulated kinase 1/2 mitogen-activated protein kinases), FAK (focal adhesion kinase), and paxillin.

**Figure 3:**
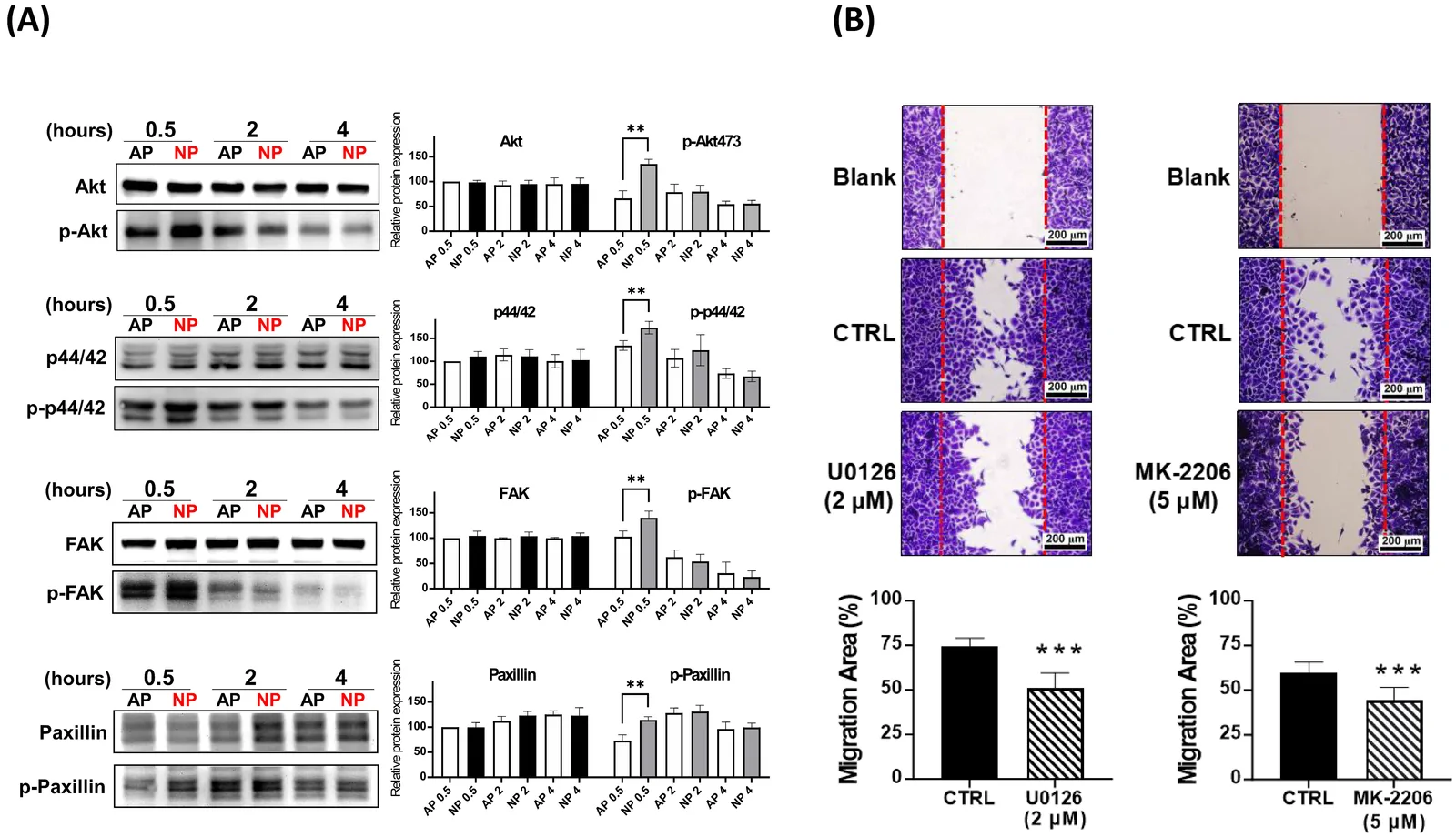
NP induces activation of AKT, ERK1/2, FAK, and paxillin in the HCEC cell line. (**A**) Western blot analysis of total and phosphorylated AKT, ERK1/2, FAK, and paxillin in the HCEC cell line at 0.5, 2, and 4 h following NP treatment (*n* = 3). (**B**) Wound healing assay demonstrating the effects of NP on migration of the HCEC cell line in the presence or absence of the AKT inhibitor MK-2206 (5 μM) and the ERK inhibitor U0126 (2 μM). Cells were pretreated with the inhibitors for 1 h before NP exposure. Migratory area was quantified after 16-h NP treatment using ImageJ (*n* = 3). Representative images are shown (original magnification ×200; scale bar, 200 μm). The red dashed lines indicate the boundaries of the initial cell-free gap. Quantitative data are presented as mean ± SEM. *** *p* < 0.001; ** *p* < 0.01. Abbreviations: Blank, baseline condition; CTRL, control.

The activation of these pathways was further evaluated by examining their phosphorylation status in the HCEC cell line following NP treatment. Western blot analysis showed that NP significantly increased the phosphorylation levels of AKT (Ser473), ERK1/2 (Thr202/Tyr204), FAK (Tyr925), and paxillin (Tyr126) as early as 0.5 h after NP exposure, whereas the total protein levels remained unchanged (Figure 3A).

To assess the functional relevance of these pathways in NP-induced cell migration, HCEC cell line was treated with U0126 (2 μM), an inhibitor of ERK1/2, and MK-2206 (5 μM), an inhibitor of AKT, for 1 h before NP exposure. Inhibition of either pathway significantly reduced NP-induced cell migration, indicating that both the ERK1/2 and AKT signaling pathways contribute to the enhanced migratory ability of HCEC cell line under NP conditions (Figure 3B).

### 2.4. NP Upregulated the Expression of G3BP1 Through Increased Protein Stability in Corneal Epithelial Cells

**Figure 4:**
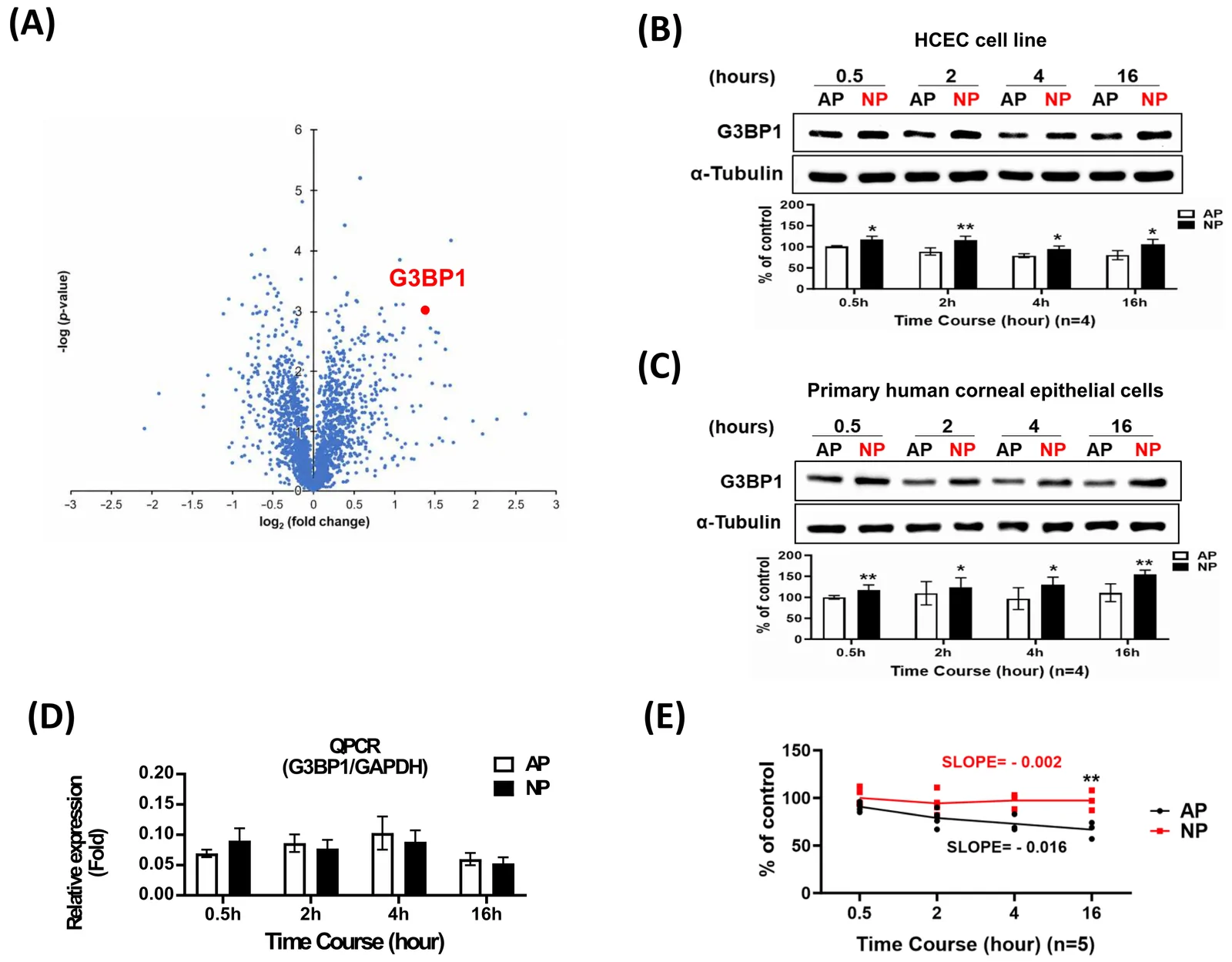
NP enhances G3BP1 expression and protein stability in corneal epithelial cells. (**A**) Volcano plot showing changes in protein expression in the membrane fraction under NP versus AP conditions. G3BP1 was significantly upregulated, with an average fold change of 2.606 in the HCEC cell line under NP treatment. (**B**) Western blot analysis of G3BP1 protein expression in HCEC cell line at 0.5, 2, 4, and 16 h after NP treatment (*n* = 4). (**C**) Western blot analysis of G3BP1 protein expression in primary human corneal epithelial cells at 0.5, 2, 4, and 16 h under AP or NP treatment (*n* = 4). (**D**) RT-qPCR analysis of G3BP1 mRNA expression in HCEC cell line at 0.5, 2, 4, and 16 h after NP treatment. (**E**) Protein stability of G3BP1 was evaluated using cycloheximide, a protein synthesis inhibitor. G3BP1 degradation was observed under AP conditions following cycloheximide treatment (*n* = 5). Representative Western blot images are provided in Supplementary Figure S3. Quantitative data are presented as mean ± SEM. * *p* < 0.05; ** *p* < 0.01.

From Volcano Plot analysis of the proteomic results, Ras GTPase activating protein SH3 domain binding protein 1 (G3BP1) was found to be one of the proteins that expressed differentially in the HCEC cell line under the NP condition. A list of the top differentially expressed proteins is provided in Table S1, while additional proteomics data are available from the corresponding author upon reasonable request. Accumulating evidence suggests that overexpression of G3BP1 is associated with tumor invasion and metastasis [28,29]. It may regulate cell migration capability by influencing processes such as cytoskeleton rearrangement, intercellular connections, and signal transduction pathways. However, research on G3BP1 in corneal epithelial cells is relatively limited, and the functional mechanisms of G3BP1 in this specific cell type have not been fully explored. Therefore, we treated the HCEC cell line with NP and found that G3BP1 exhibited a significant increase under NP conditions, with an average fold change of 2.606 after NP treatment. This significant increase in expression suggests that G3BP1 is highly responsive to NP conditions, potentially playing a key role in the cellular response or physiological changes induced by NP treatment (Figure 4A). The protein expression of G3BP1 in cells at 0.5, 2, 4 and 16 h after NP conditions was further determined by Western blot analysis. The results confirmed that the G3BP1 protein expressed significantly higher at all four time points after NP treatment compared to those in the AP condition (Figure 4B). To strengthen these findings, we also analyzed G3BP1 expression in primary human corneal epithelial cells under the same conditions. Consistently, G3BP1 protein levels were markedly upregulated in response to NP at all time points (Figure 4C). The mRNA expression of G3BP1 at 0.5, 2, 4 and 16 h after NP conditions was measured by RT qPCR analysis, but no significant difference was found (Figure 4D).

To explore the mechanism underlying this protein increase, we assessed G3BP1 protein stability by treating cells with cycloheximide, an inhibitor of protein synthesis. Under AP conditions, G3BP1 protein levels decreased progressively after cycloheximide treatment. However, under NP conditions, G3BP1 degradation was delayed, and the protein remained stable during the first 2 h (* *p* < 0.05, Figure 4E). These findings suggest that NP enhances G3BP1 protein stability, rather than its transcription, thereby contributing to its accumulation.

### 2.5. Knockdown of G3BP1 Markedly Reduced Cell Migration and Enhanced Expression of E-Cadherin, p120 and ZO-1 in Corneal Epithelial Cells Under NP Conditions

G3BP1 has been implicated in the regulation of cell migration in various cancer types through its interaction with Ras-GAP and the modulation of signaling pathways such as Ras and Wnt/β-catenin, which play roles in tumor progression and metastasis [24,27]. However, the involvement of G3BP1 in corneal epithelial cells, particularly in relation to NP-induced migration, has not been well studied.

**Figure 5:**
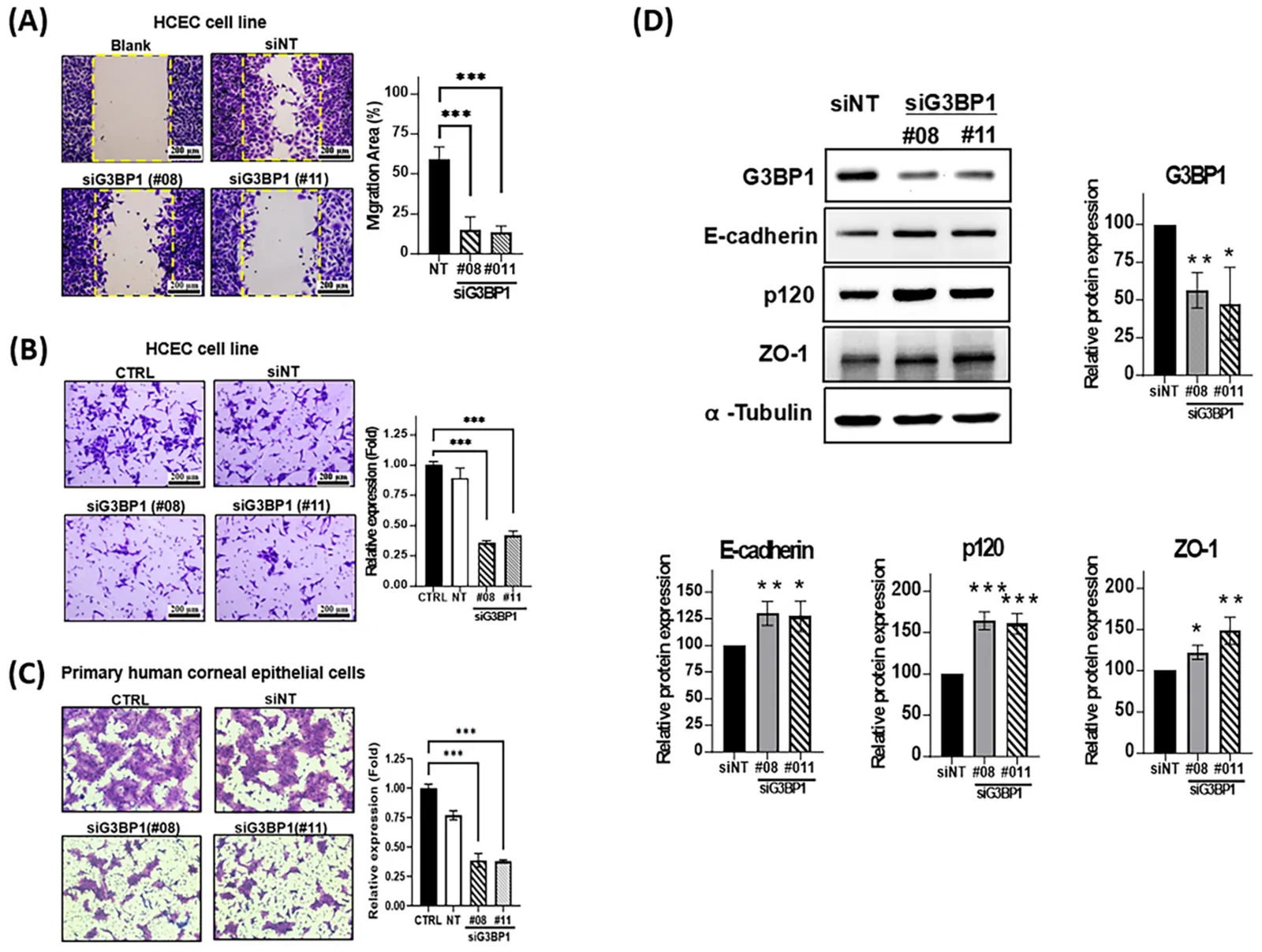
Effects of G3BP1 knockdown on corneal epithelial cell migration and membrane protein expression. (**A**) Wound healing assay showing migration of the HCEC cell line following G3BP1 knockdown. Cells were transfected with G3BP1-specific siRNAs (siG3BP1 #08 and #11) or negative control siRNA (siNT). Migratory area was quantified using ImageJ. Representative images are shown (original magnification ×200; scale bar, 200 μm). Abbreviations: Blank, untreated HCEC cell line; siNT, negative control siRNA; siG3BP1 (#08, #11), siRNA targeting G3BP1 (ID: n278908, n278911). (**B**) Transwell migration assay of the HCEC cell line following G3BP1 knockdown. Cells were transfected with siG3BP1 (#08 and #11) or siNT, and migration was assessed after incubation (*n* = 3). (**C**) Transwell migration assay of primary human corneal epithelial cells following G3BP1 knockdown under the same conditions. In both cell types, G3BP1 knockdown significantly impaired cell migration compared with siNT controls. Representative images are shown (original magnification ×200; scale bar, 200 μm). Abbreviations: Control, untreated control; siNT, negative control siRNA; siG3BP1 (#08, #11), siRNA targeting G3BP1 (ID: n278908, n278911). (**D**) Western blot analysis of membrane-associated proteins, including E-cadherin (135 kDa), p120 (110 kDa), and ZO-1 (225 kDa), in the HCEC cell line following G3BP1 knockdown (*n* = 3). Quantitative data are presented as mean ± SEM. * *p* < 0.05; ** *p* < 0.01; *** *p* < 0.001. The G3BP1 blot shown in this panel was derived from the same protein lysates as those presented in Figure 6. Cells in the representative images in (**A**–**C**) were stained with crystal violet (purple).

In this study, we knocked down the expression of G3BP1 in corneal epithelial cells using the siRNA method and then analyzed cell migration and the expressions of E-cadherin, ZO-1 and p120 in the HCEC cell line. Two different siRNAs (n278908 and n278911; marked as siG3BP1 #08 and siG3BP1 #11) were used in this study to knock down G3BP1. G3BP1 knockdown markedly suppressed cell migration, with an approximately 85% reduction in wound closure compared with the siNT control, as demonstrated by the wound healing assay (*** *p* < 0.001; Figure 5A). This inhibitory effect was further confirmed using a Transwell migration assay, in which G3BP1 knockdown reduced cell migration by 58–64% relative to the siNT control (Figure 5B). Similarly, a comparable reduction in migratory ability was observed in primary human corneal epithelial cells following G3BP1 knockdown (Figure 5C), supporting the critical role of G3BP1 in promoting corneal epithelial cell migration across different cell sources. In addition, G3BP1 knockdown increased the expression levels of E-cadherin, p120-catenin, and ZO-1 in the HCEC cell line, as determined by Western blot analysis (Figure 5D). These findings suggest that the NP-induced cell migration and the suppressed expressions of E-cadherin, p120 and ZO-1 in corneal epithelial cells are likely mediated by the upregulation of G3BP1 expression.

### 2.6. G3BP1 Knockdown Suppressed the Activation of AKT/ERK/FAK/Paxillin Pathways in Corneal Epithelial Cells

**Figure 6:**
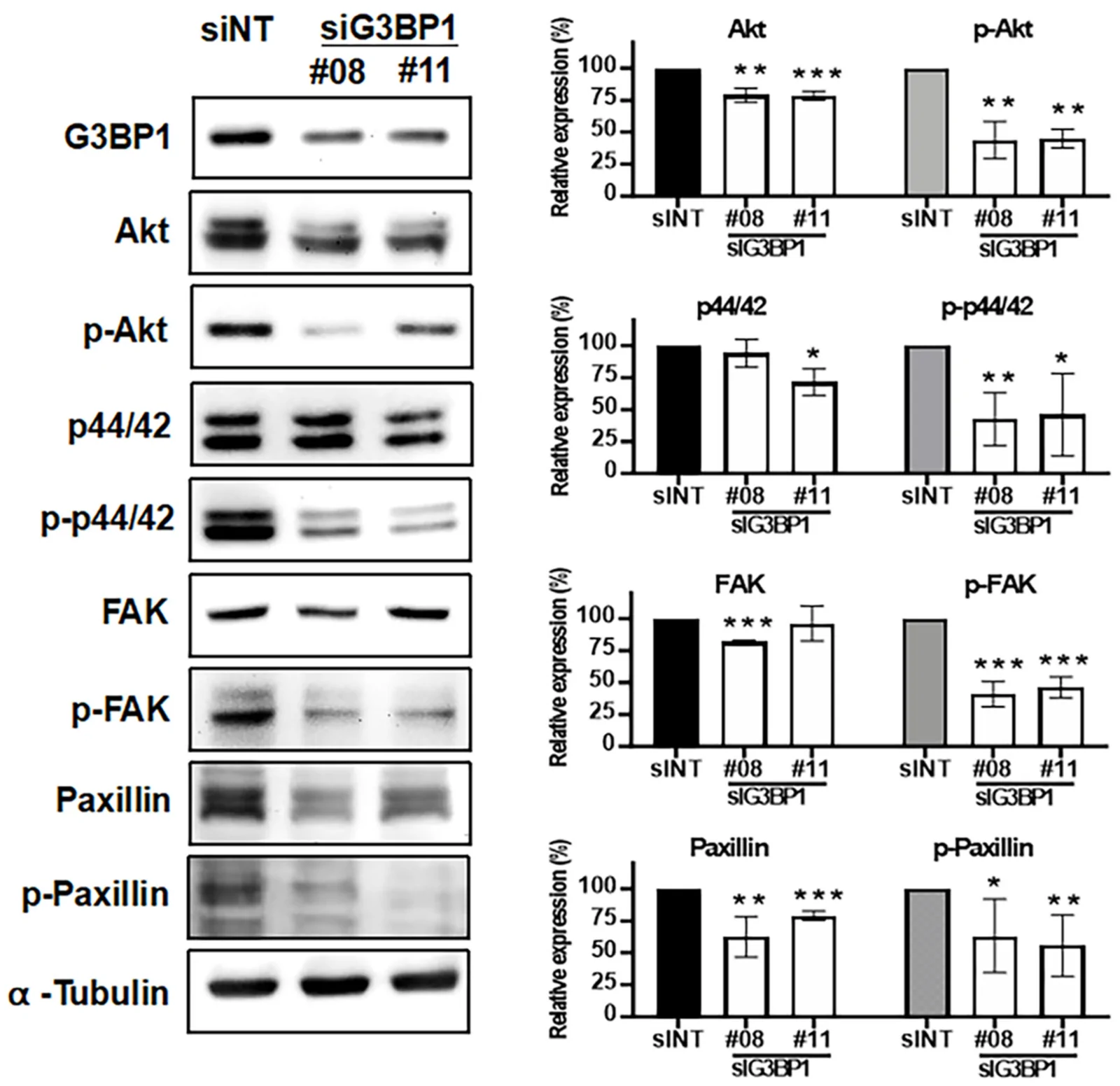
Effect of G3BP1 knockdown on AKT/ERK1/2/FAK/Paxillin signaling pathways in the HCEC cell line. Western blot analysis was performed to evaluate total and phosphorylated levels of AKT, ERK1/2, FAK, and paxillin in the HCEC cell line following G3BP1 knockdown using siRNA. The effects of G3BP1 silencing on these signaling pathways were quantified (*n* = 3). Representative data show changes in protein expression and phosphorylation after transfection with G3BP1-specific siRNAs (siG3BP1 #08 and #11) compared with negative control siRNA (siNT). Quantitative data are presented as mean ± SEM. * *p* < 0.05; ** *p* < 0.01; *** *p* < 0.001. Abbreviations: siNT, negative control siRNA; siG3BP1 (#08, #11), siRNA targeting G3BP1 (ID: n278908, n278911). The G3BP1 blot shown in this figure was derived from the same protein lysates obtained from a single G3BP1 knockdown experiment as those presented in Figure 5D and is included here to confirm knockdown efficiency in the signaling analysis.

To further investigate the role of G3BP1 in the activation of AKT, ERK1/2 MAP kinases, FAK, and paxillin in the HCEC cell line, we knocked down the expression of G3BP1 in corneal epithelial cells using the siRNA method and analyzed the levels of these proteins and their phosphorylation states. The results demonstrated that G3BP1 knockdown not only reduced the phosphorylation of AKT, ERK, and paxillin but also significantly decreased their total protein expression levels (Figure 6). The findings suggest that these signaling pathways may play a role in corneal epithelial cell migration induced by G3BP1.

## 3. Discussion

In this study, for the first time, we investigated the effects of NP on corneal epithelial wound healing. We demonstrated that NP promotes corneal epithelial cell migration without affecting cell viability through the attenuation of membrane-associated junction proteins, including E-cadherin, p120, and ZO-1. Furthermore, we found that the AKT/ERK/FAK/paxillin signaling pathways are critically involved in NP-induced HCEC migration. G3BP1 plays a pivotal role in regulating membrane-to-cytosol signaling associated with corneal epithelial wound healing.

The process of corneal epithelial wound healing is highly complex, involving cellular proliferation, migration, apoptosis, and extracellular matrix remodeling, which restore the integrity and transparency of the corneal epithelium. Dysregulated healing may lead to PCEDs, significantly impacting visual outcomes [13]. Current clinical treatments, including bandage contact lenses [30], autologous serum eye drops [31], and amniotic membrane transplantation, have limitations, such as risks of infection, corneal opacification, and high recurrence rates in PCEDs [32]. Therefore, there is an urgent need for innovative therapeutic strategies to enhance corneal wound healing efficiently and safely.

According to the experimental observations, negative pressure has multiple effects on corneal epithelial cells. Firstly, it decreases the levels of cell junction and adhesive molecules, potentially leading to destabilized cell junctions and facilitating cell migration. Although reduced expression of E-cadherin, p120, and ZO-1 may transiently compromise epithelial barrier integrity, the present study focused primarily on the early molecular events associated with NP-induced epithelial migration during short-term stimulation. Restoration of epithelial barrier function following wound closure warrants further investigation. Additionally, negative pressure induces changes in cell structure and protein composition, particularly between the cell membrane and cytosol. These alterations suggest that the structure and composition of the cell membrane may be affected, which could also impact processes such as intracellular solute transport and cell signaling. The results obtained from tandem mass spectrometry analysis reveal that under negative-pressure conditions, the functionality of most cell membrane proteins is reduced, while the functionality of most cytosol proteins is enhanced. In our study, we conducted a time-course analysis to examine the effects of negative pressure on cellular responses. Through proteomic analysis, we observed changes in protein expression over different time points following negative-pressure treatment. Interestingly, we identified a candidate protein molecule, G3BP1, which exhibited significant temporal differences in expression compared to normal pressure conditions. Quantitative analysis revealed that negative pressure upregulated the expression of G3BP1 and resulted in a prolonged stability of G3BP1 protein, indicating a potential role in the cellular response to negative pressure. According to previous studies, G3BP1 protein is an SH3-binding protein 1 for Ras GTPase-activating protein and is involved in the expression of various tumor occurrences and metastases [24,33,34]. A study in 2013 demonstrated that downregulation of G3BP protein can inhibit the phosphorylation of Src, FAK, and ERK, leading to a decrease in levels of MMP-2, MMP-9, and uPA, further suppressing the migration and invasion of lung cancer cells [25]. In the 2023 ARVO journal abstracts, it was also mentioned that researchers have found that G3BP1/2 proteins play a crucial role in corneal wound healing and may become new targets for anti-scarring therapies in the cornea [35].

To delve deeper into the functional significance of G3BP1, we conducted knockdown experiments. Notably, silencing G3BP1 not only stabilized the expression of E-cadherin/p120/ZO-1 adhesion proteins but also downregulated the expression of AKT/ERK/Paxillin, resulting in a slowdown of cell migration. This finding confirms the downregulation of cell adhesion and adhesive molecules on the cell membrane in response to negative pressure, which is achieved through the early phosphorylation of AKT/ERK/Paxillin, thereby promoting increased cell motility. Previous studies have also demonstrated that ERK regulates cell migration during in vitro wound healing by modulating the phosphorylation of FAK and focal adhesion proteins [36]. In 2019, researchers confirmed that knocking down G3BP1 protein can inhibit the proliferation and migration of esophageal cancer cells through the Wnt/β-catenin and PI3K/AKT signaling pathways [24]. The study also indicated that overexpression of G3BP1 protein promotes cell proliferation, apoptosis, and development by activating β-catenin, while knocking down G3BP1 protein inhibits cell migration and invasion [28]. After G3BP1 knockdown, significant changes were observed in the expression of epithelial–mesenchymal transition markers such as E-cadherin, Snail, and vimentin, leading to inhibition of cell migration. Furthermore, upon G3BP1 knockdown, not only was the phosphorylation of AKT/ERK/Paxillin downregulated [29], but GSKα/β was also inhibited [37], which is involved in mediating the Akt pathway. In the present study, phosphorylation was quantified as the ratio of phosphorylated protein to its corresponding total protein. Nevertheless, because G3BP1 knockdown also reduced the abundance of several total signaling proteins, we cannot completely exclude the possibility that G3BP1 influences both pathway activation and overall protein expression. Therefore, future rescue experiments using siRNA-resistant G3BP1 will be valuable to further clarify the direct mechanistic relationship.

**Figure 7:**
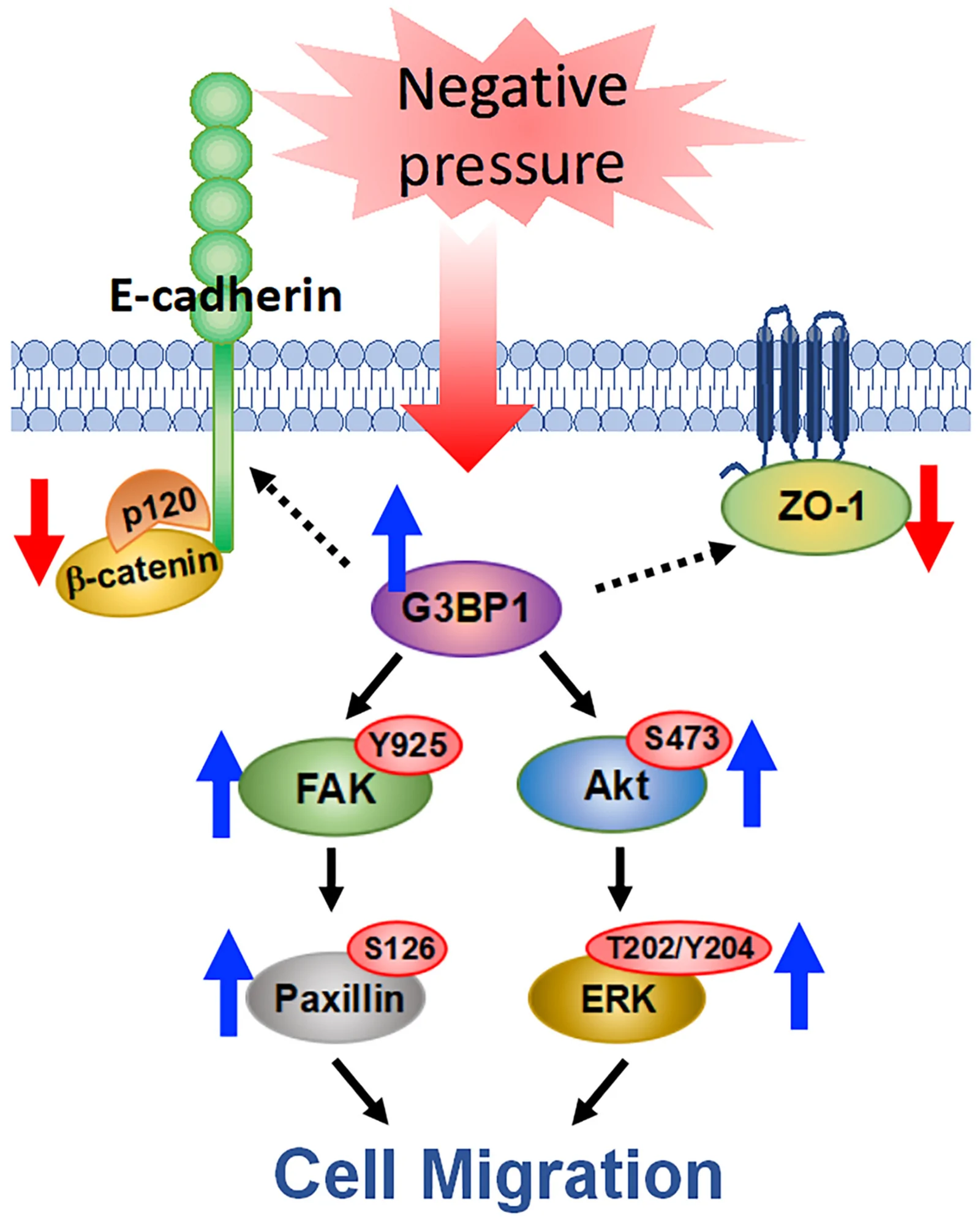
Proposed molecular mechanisms underlying negative pressure-induced migration of corneal epithelial cells. Negative pressure (NP) promotes corneal epithelial cell migration through coordinated regulation of adhesion molecules and intracellular signaling pathways. NP induces downregulation of membrane-associated adhesion proteins, including E-cadherin, p120-catenin, and ZO-1, thereby weakening cell–cell junctions and facilitating cell motility. Concomitantly, NP upregulates G3BP1 expression in the HCEC cell line, which is associated with activation of key signaling pathways involved in cell migration, including AKT, ERK1/2, FAK, and paxillin. Activation of these pathways enhances cytoskeletal dynamics and promotes a pro-migratory cellular phenotype. Collectively, these findings support a role for G3BP1 in the signaling network associated with NP-induced corneal epithelial wound healing. In the schematic, blue upward arrows indicate increased expression or activation, red downward arrows indicate decreased expression, black solid arrows indicate proposed signaling relationships, and black dashed arrows indicate proposed regulatory relationships.

According to our findings, negative pressure reduced the expression of cell adhesion molecules and promoted corneal epithelial cell migration. We identified G3BP1 as a candidate regulator that was markedly upregulated under negative-pressure conditions. G3BP1 upregulation was associated with reduced E-cadherin expression and activation of the AKT/ERK/FAK/paxillin signaling pathways, suggesting that G3BP1 contributes to the molecular regulation of corneal epithelial migration under negative pressure (Figure 7).

Considering these findings, it is hoped that through negative-pressure treatment and identifying candidate proteins, we can shed light on identifying novel targets to promote corneal epithelial wound healing. Although the present study identified G3BP1-mediated signaling pathways involved in negative pressure-induced corneal epithelial migration, future studies using single-cell RNA sequencing (scRNA-seq) may provide additional insights into cellular heterogeneity and dynamic intracellular and intercellular signaling during corneal wound healing. Integrating scRNA-seq with functional analyses may further validate the molecular mechanisms identified in the present study and facilitate the development of more precise therapeutic strategies [38,39]. Although negative pressure therapy has shown promising results in other fields, further research is needed to evaluate its effectiveness and safety in the treatment of ocular surface diseases. The unique characteristics of ocular tissues and the ocular environment may have an impact on the application of negative pressure therapy. Therefore, additional experiments and clinical trials are mandatory to determine the feasibility and efficacy of negative pressure therapy for wound healing in ocular surface diseases.

## 4. Materials and Methods

### 4.1. Negative Pressure Chamber Setup

The negative pressure chamber (NPI-2000, Linston Advanced Technology, Longtan, Taiwan [23]) was designed to maintain controlled environmental conditions during negative-pressure exposure. In this study, a target pressure of −125 mmHg was applied continuously throughout the indicated treatment periods. A vacuum pump (46 L/min) reduced the chamber pressure at a rate of approximately 12.5 mmHg/s, and the preset pressure was adjusted using a manual control knob. Chamber pressure was continuously monitored using both an electronic pressure gauge and a mechanical pressure gauge to ensure accurate and stable pressure throughout the experiments.

To maintain physiological culture conditions, ambient air was supplied to maintain approximately 20% oxygen, and a continuous CO_2_ supply maintained 5% CO_2_. Oxygen and CO_2_ concentrations were continuously monitored using a gas analyzer. Chamber temperature was maintained at 37 °C using an electronic temperature control system and verified with an independent thermometer placed inside the chamber. Humidity was maintained by placing a water reservoir inside the chamber throughout the experiments. Atmospheric-pressure (AP) control cells were cultured in a standard CO_2_ incubator under the same temperature, CO_2_ concentration, and humidity conditions. A schematic illustration of the experimental setup is shown in Figure S1.

### 4.2. Cell Culture

The HCEC cell line (CRL-11515, American Type Culture Collection, ATCC, Manassas, VA, USA) used in this study was obtained in 2017 from the laboratory of Dr. Wei-Li Chen at the Department of Ophthalmology, National Taiwan University Hospital [40]. Cells were maintained in keratinocyte serum-free medium (KSFM; #17005-042, Gibco, Thermo Fisher Scientific, Waltham, MA, USA) supplemented with 5 ng/mL recombinant human epidermal growth factor (rhEGF), 0.05 mg/mL bovine pituitary extract, 0.005 mg/mL insulin, and 500 ng/mL hydrocortisone.

Primary human corneal epithelial cells were isolated residual from limbal tissue obtained from human corneas after corneal transplantation procedures and cultured in supplemental hormonal epithelial medium (SHEM), which was prepared using DMEM/F12 medium (#12400-024, Gibco, Thermo Fisher Scientific, Waltham, MA, USA) containing 5% fetal bovine serum (FBS), 50 μg/mL gentamicin, 5 μg/mL insulin, 5 ng/mL selenium, 1.25 μg/mL amphotericin B, 5 μg/mL transferrin, 2 ng/mL mouse EGF, 0.5 μg/mL hydrocortisone, 30 ng/mL cholera toxin, and 0.5% dimethyl sulfoxide (DMSO). DMEM/F12, FBS, and mouse EGF were purchased from Gibco, while other reagents were obtained from Sigma-Aldrich (St. Louis, MO, USA).

The use of human-derived tissues was approved by the Institutional Review Board of Chang Gung Memorial Hospital (IRB No. 201802354A3B0). All procedures adhered to the tenets of the Declaration of Helsinki. The requirement for informed consent was waived by the Institutional Review Board because residual human tissues were used in this study.

Both the HCEC cell line and primary human corneal epithelial cells were cultured in a humidified incubator at 37 °C with 5% CO_2_ and 95% air. Primary human corneal epithelial cells were used at passage 2 (P2) for all experiments, whereas the HCEC cell line was routinely maintained in our laboratory and used between passages 6 and 15. Both the HCEC cell line and primary human corneal epithelial cells were passaged at confluence using 0.05% trypsin-EDTA. Prior to experimental treatments, cells were subcultured for 24 h and subsequently exposed to either atmospheric-pressure (AP) or negative-pressure (NP) conditions as described in the experimental design. Cells were harvested at the indicated time points for subsequent analyses. Primary human corneal epithelial cells were established from approximately 10–12 independent donors. Because each donor sample yielded a limited number of cells, individual experiments were performed using cells from 4–5 independent donors, and each donor sample was used only once for a given analysis.

### 4.3. Cell Migration Assay

Cell migration was analyzed by two different methods. To create a 500 µm cell-free gap, the SPLScar^TM^ Block (#21902, SPL Life Sciences, Seoul, Republic of Korea) was applied, generating the gap by removing a silicone insert from a confluent culture of human corneal epithelial cells. After exposure to AP or NP conditions for 16 h, cells were stained with 0.1% Coomassie blue. The stained regions within the cell-free gap were captured using a digital microscope, and the migration area was quantified using ImageJ software (version 1.54g; NIH, Bethesda, MD, USA). The percentage of gap closure was calculated by comparing the initial gap area at 0 h with the remaining unstained area after 16 h of treatment. This quantitative analysis allowed for precise measurement of cell migration into the gap. Migration was also evaluated using a transwell migration assay. Cells at 2 × 10^5^ cells/150 µL culture medium per well were seeded in upper chamber of transwell plate fitted with 8 μm pore-size filters (#37224, SPL Life Sciences, Seoul, Republic of Korea), while 750 μL culture medium was added to the lower chamber. The transwell plate was placed under AP or NP conditions for 16 h. After incubation, cells that had migrated through the transwell filter were stained with 0.5% crystal violet. The filters were then gently rinsed with PBS to remove excess dye and allowed to dry. Quantitative analysis was conducted by manually counting the stained cells on the underside of the filter under a light microscope at 100× magnification in five randomly selected fields per filter. The average cell count from these fields was calculated for statistical analysis.

### 4.4. Cell Viability Assay

Cell viability was measured by the Cell Counting Kit-8 (CCK-8) assay (Cat. No. 96992, Sigma-Aldrich, St. Louis, MO, USA). The HCEC cell line and primary human corneal epithelial cells (1 × 10^4^ cells per 100 μL per well) were seeded into a 96-well plate (#3599, Corning Costar, NY, USA) and cultured for 24 h under standard conditions. Cells were then subjected to either AP or NP conditions for 16 h. After the treatment, 10 μL of CCK-8 solution was added to each well and incubated at 37 °C in a humidified atmosphere containing 5% CO_2_ for 3 h. The absorbance at 450 nm was measured using a microplate reader to quantify cell viability. Data were recorded and analyzed to determine the effects of AP and NP conditions on corneal epithelial cell viability.

### 4.5. Preparation of Cell Lysates and Western Blot Analysis

The HCEC cell line and primary human corneal epithelial cells exposed to AP or NP conditions for 16 h were washed twice with ice-cold phosphate-buffered saline (PBS) and lysed in a buffer containing 20 mM Tris–HCl, 50 mM β-glycerol phosphate, 100 mM NaCl, 1 mM Na_3_VO_4_, 1 mM Na_4_P_2_O_7_, 0.5 mM EDTA, 1 mM EGTA, 1% Triton X-100, 1 mM DTT, 0.5 mM PMSF, 2 µg/mL aprotinin, 2 µg/mL leupeptin, and 2 µg/mL pepstatin (all reagents from Sigma-Aldrich, St. Louis, MO, USA). The lysates were centrifuged, and the supernatants were collected for protein analysis. Protein concentrations were determined using the bicinchoninic acid (BCA) assay kit (#23227, Thermo Fisher Scientific, Waltham, MA, USA), with bovine serum albumin (BSA) as the standard.

Proteins were separated on 8–12% SDS-PAGE gels and transferred onto polyvinylidene fluoride (PVDF) membranes (IPVH00010, Millipore, Burlington, MA, USA). Membranes were blocked in blocking buffer and incubated overnight at 4 °C with primary antibodies diluted in the blocking buffer. The following primary antibodies were used: E-cadherin (1:2000, cs-3195), Akt (1:1000, cs-4691), phospho-Akt473 (1:1000, cs-4060), p44/42 MAPK (1:2000, cs-4695s), phospho-p44/42 MAPK (1:1000, cs-4370), FAK (1:2000, cs-3285), phospho-FAK (Tyr925) (1:1000, cs-3284), G3BP1 (1:2000, cs-61559s) (both from Cell Signaling Technology, Danvers, MA, USA). p120 (1:2000, CM3541, ECM Biosciences, Versailles, KY, USA), α-tubulin (1:5000, #05-829), phospho-paxillin (Tyr126) (1:2000, #07-1441) (both from Millipore, Burlington, MA, USA), ZO-1 (1:2000, #33-9100), and paxillin (1:500, MA5-13356) (both from Thermo Fisher Scientific, Waltham, MA, USA).

After washing with TBST three times for 15 min each, the membrane was incubated for 1 h with secondary antibodies: anti-rabbit (1:3000, Cat. No. 314430) or anti-mouse (1:3000, Cat. No. 314460) (both from Thermo Fisher Scientific, Waltham, MA, USA). The immunoreactive bands were detected by Immobilon ECL Ultra HRP substrate (WBULS0500, Millipore, Burlington, MA, USA) and quantitated using UVPBioSpectrum 810 Imaging System with VisionWorksLS Image Acquisition and Analysis Software (version 8.6.15114.8618, UVP, Upland, CA, USA).

Western blot analyses for multiple targets were performed using the same protein lysates derived from a single G3BP1 knockdown experiment to ensure consistency across analyses.

### 4.6. Immunofluorescent Staining

The HCEC cell line and primary human corneal epithelial cells were seeded onto glass coverslips and cultured for 24 h prior to treatment under AP or NP conditions for 16 h. Cells were fixed in 100% methanol (Merck Millipore, Darmstadt, Germany) at −20 °C for 15 min and permeabilized with 0.1% Triton X-100 in PBS for 10 min. Unless otherwise noted, general reagents were purchased from Sigma-Aldrich (St. Louis, MO, USA). Coverslips were incubated with primary antibodies diluted 1:200 overnight at 4 °C, washed three times with PBS for 5 min each, and then incubated with DyLight^®^ 488-conjugated secondary antibodies (Thermo Fisher Scientific, Waltham, MA, USA) diluted 1:500 for 1 h at room temperature in the dark. The antibodies used included E-cadherin (cs-3195, Cell Signaling Technology, Danvers, MA, USA), p120 (CM3541, ECM Biosciences, Versailles, KY, USA), ZO-1 (#33-9100, Thermo Fisher Scientific), goat anti-rabbit IgG DyLight^®^ 488 (#A120-201D2, Bethyl Laboratories, Montgomery, TX, USA), and donkey anti-mouse IgG DyLight^®^ 488 (#A90-337D2, Bethyl Laboratories). Nuclei were counterstained with 4′,6-diamidino-2-phenylindole (DAPI) (Thermo Fisher Scientific). Following staining, coverslips were washed, mounted onto glass slides using antifade mounting medium, and visualized under a Leica TCS SP8X confocal microscope (Leica Microsystems, Wetzlar, Germany).

### 4.7. Knockdown of Gene Expression by Small Interfering RNA (siRNA)

Cells were seeded at a density of 1.5 × 10^5^ cells per well in a 6-well plate without antibiotics, ensuring 30–50% confluency at the time of transfection. In this study, two siRNAs (n278908 and n278911) targeting different regions of G3BP1 mRNA were used to ensure high specificity and to minimize off-target effects. These siRNAs were tested separately to evaluate their individual effects on G3BP1 expression and downstream signaling pathways.

The siRNA-Lipofectamine™ RNAiMAX complexes were prepared by mixing 30 pmol siRNA (ID: n278908 and n278911, Thermo Fisher Scientific, Waltham, MA, USA) with 150 µL Opti-MEM™ Medium (#31985062, Gibco, Thermo Fisher Scientific, Waltham, MA, USA) and 9 µL Lipofectamine™ RNAiMAX reagent (#13778150, Invitrogen, Thermo Fisher Scientific, Waltham, MA, USA) in 150 µL Opti-MEM™ Medium. The mixture was incubated at room temperature for 5 min before being added dropwise to each well. Cells were incubated with the transfection complexes for three days in a 37 °C humidified incubator with 5% CO_2_ and 95% air.

### 4.8. Proteomics Analysis

Cells were harvested at 0.5, 2, 4, and 16 h after treatment under AP or NP conditions. Subcellular fractions, including membrane, cytoplasm, and nucleus, were separated using a subcellular protein fractionation kit (#78840, Thermo Fisher Scientific, Waltham, MA, USA). Quantitative proteomics analysis was performed using Tandem Mass Tag (TMT) labeling kits (6-plex) (#90066, Thermo Fisher Scientific, Waltham, MA, USA), which involved disulfide bonding reduction, thiol group alkylation, enzyme digestion, and tag labeling. Briefly, proteins were reduced with 20 mM TCEP for 1 h at 55 °C, alkylated with 35 mM iodoacetamide for 30 min in the dark at room temperature, acetone-precipitated with 50 mM TEAB, and enzymatically digested with trypsin at 37 °C overnight. Subsequently, these samples were labeled with TMT reagents and mixed before sample fractionation and cleanup.

The TMT-labeled peptides were analyzed by nano-LC-ESI-MS on an Orbitrap LUMOS mass spectrometer (Thermo Fisher Scientific) equipped with an Ultimate 3000 nanoLC system (Thermo Fisher Scientific) and a nano-electrospray ion source (New Objective, Woburn, MA, USA). A 4 μL peptide solution was separated on a 75-μm internal diameter, 25-cm length C18 Acclaim PepMap NanoLC column (Thermo Fisher Scientific) packed with 2 μm particles and a pore size of 100 Å, using 0.1% formic acid in water (mobile phase A) and 0.1% formic acid in 80% acetonitrile (mobile phase B), operated at a 300 nL/min flow rate. The LC gradient employed was 5% buffer B at 2 min to 40% buffer B at 90 min. The full-scan MS was operated in a mass range of *m*/*z* 350 to 1600 with a resolution of 120,000 and a maximum injection time of 50 ms. The target *m*/*z* was isolated and subjected to data-dependent MS/MS scan by HCD with assisted NCE mode at 25, 35, and 45, with a 15,000 resolution and a 50 ms maximum injection time. Electrospray voltage was maintained at 1.8 kV and the capillary temperature at 275 °C.

The acquired MS raw data were analyzed using MaxQuant (version 1.5.3.30, Max Planck Institute of Biochemistry, Martinsried, Germany) with the human protein database obtained from UniProt reviewed human proteomes for TMT labeling quantitation. For database searches, the precursor mass tolerance was set to 20 ppm for the first searches and 4.5 ppm for the main database search. The fragment ion mass tolerance was set to 0.5 Da. Trypsin was chosen as the enzyme with 2 missed cleavages allowed. Carbamidomethylation of cysteine was defined as a fixed modification and oxidation of methionine as a variable modification. Minimum peptide length was set to seven amino acids. The maximum false-discovery rate, calculated using a reverse database, was set to 1% for both peptides and proteins. Proteins identified as “reverse” and “only identified by site” were excluded from the list of identified proteins.

### 4.9. Quantitative RT-PCR

Total RNA was extracted from HCEC cell line using TRIzol reagent (Invitrogen, Thermo Fisher Scientific, Waltham, MA, USA) according to the manufacturer’s instructions. Complementary DNA (cDNA) was synthesized using SuperScript III Reverse Transcriptase (Invitrogen, Thermo Fisher Scientific) following the manufacturer’s protocol. Quantitative real-time PCR was performed using gene-specific primers for G3BP1 and GAPDH. Relative gene expression levels were normalized to GAPDH and calculated using the 2^−ΔΔCt^ method. The primer sequences used were as follows: G3BP1 forward, 5′-TGAGGTCTTTGGTGGGTTTG-3′; G3BP1 reverse, 5′-TGCTGTCTTTCTTCAGGTTCC-3′; GAPDH forward, 5′-CCACTAGGCGCTCACTGTTCTC-3′; and GAPDH reverse, 5′-ACTCCGACCTTCACCTTCCC-3′.

### 4.10. Cycloheximide Chase Assay

To evaluate G3BP1 protein stability, the HCEC cell line was treated with cycloheximide (50 μg/mL; Sigma-Aldrich, St. Louis, MO, USA), an inhibitor of protein synthesis, for 1 h under AP or NP conditions. Protein samples were collected at the indicated time points and analyzed by Western blotting. Band intensities were quantified using the UVP imaging system after normalization to α-tubulin.

### 4.11. Statistical Analysis

All quantitative data were analyzed with GraphPad Prism 8 software (GraphPad Software, San Diego, CA, USA). The area of cell migration was quantified using ImageJ software (National Institutes of Health, Bethesda, MD, USA). Unless otherwise indicated, each experiment was performed with at least three independent biological replicates, and n represents independent biological experiments. For experiments using primary human corneal epithelial cells, n represents independent donor samples. Statistical analyses were conducted using one-way ANOVA to compare differences among multiple groups, and Student’s *t*-test was applied only for comparisons between two groups. Data are presented as mean ± standard error of the mean (SEM), and a *p* value < 0.05 was considered statistically significant.

## 5. Conclusions

In conclusion, the present study provides the first comprehensive investigation of the molecular mechanisms underlying negative pressure (NP)-induced corneal epithelial wound healing. We demonstrated that NP promotes corneal epithelial cell migration without affecting cell viability and induces dynamic remodeling of epithelial junction proteins, including E-cadherin, p120, and ZO-1, thereby facilitating cell motility. Proteomic analysis further revealed that NP alters the subcellular distribution of proteins between the membrane and cytosol, suggesting that membrane remodeling is an early cellular response to NP stimulation.

Mechanistically, NP rapidly activated the AKT/ERK/FAK/paxillin signaling pathways and increased the protein stability of G3BP1 without significantly altering its mRNA expression, indicating that post-translational regulation contributes to the NP response. Functional studies further demonstrated that G3BP1 knockdown markedly attenuated NP-induced cell migration, restored junctional protein expression, and suppressed activation of the AKT/ERK/FAK/paxillin signaling cascade, supporting an important role for G3BP1 in the signaling network associated with NP-induced corneal epithelial migration.

Collectively, our findings support a model in which NP promotes corneal epithelial migration through coordinated regulation of junctional remodeling and intracellular signaling, with G3BP1 serving as a key mediator linking extracellular mechanical stimulation to downstream signaling events. These findings provide new insights into the molecular mechanisms underlying corneal epithelial wound healing and establish a biological basis for future translational studies investigating NP-based therapeutic strategies. Further in vivo studies are warranted to evaluate the safety, feasibility, and therapeutic potential of ocular NP application before clinical translation.

## Data Availability

The original contributions presented in this study are included in the article and Supplementary Materials. Further inquiries can be directed to the corresponding authors.

## Supplementary Materials

The following supporting information can be downloaded at https://www.mdpi.com/article/10.3390/ijms27167273/s1.
