# Supplementary material for: Negative Pressure Promotes G3BP1-Mediated Migration of Corneal Epithelial Cells Through Activation of AKT/ERK/Paxillin Pathway
Source: Int J Mol Sci. 2026 Aug 14;27(16):7273. doi: 10.3390/ijms27167273 (PMC13513308; doi:10.3390/ijms27167273)
Supplement: Supplementary file 1 [file ijms-27-07273-s001.zip › ijms-4371092-supplementary.pdf]

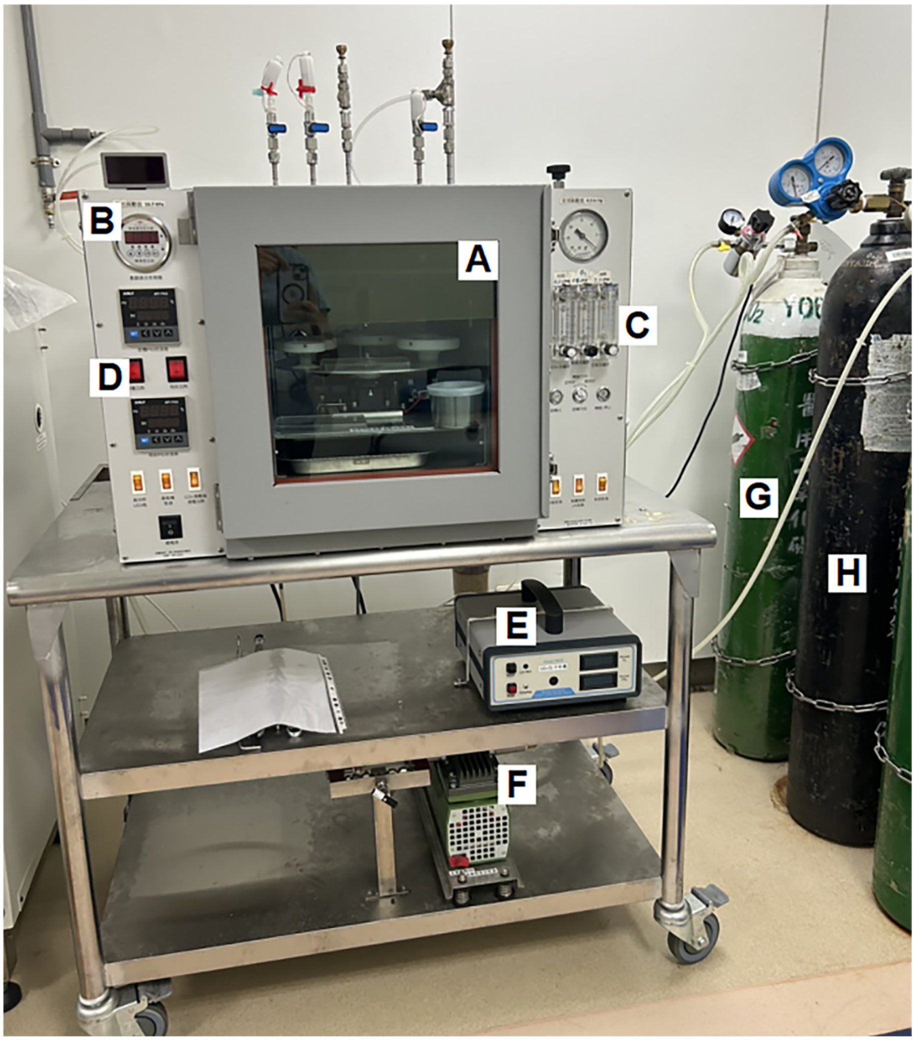

Figure S1. Negative pressure chamber setup (**A**–**H**).

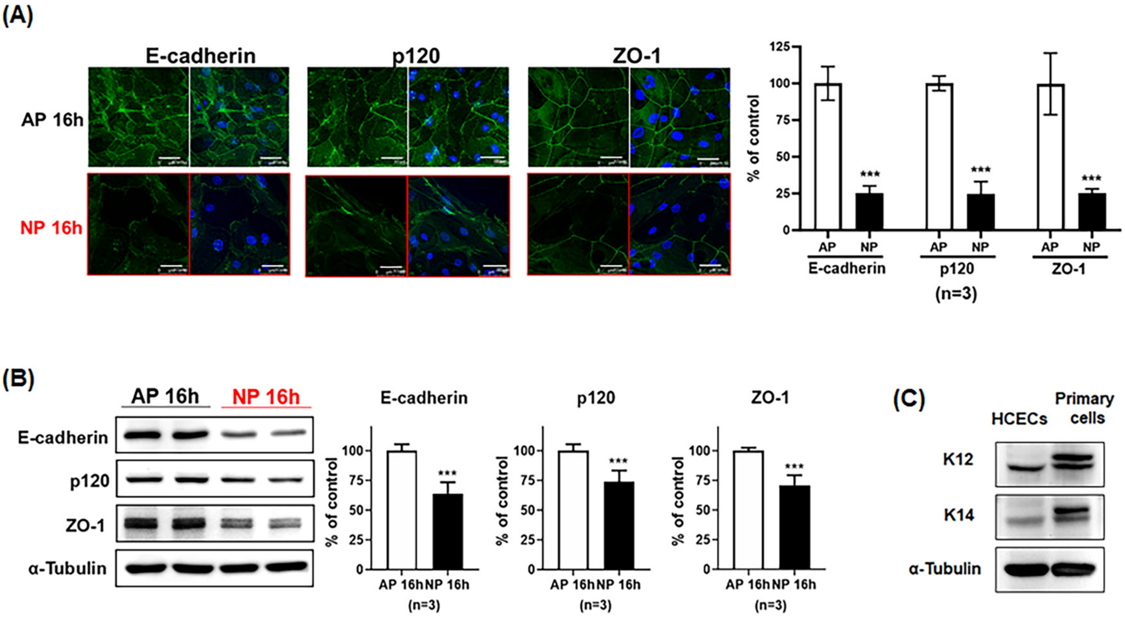

Figure S2. (**A**,**B**) Effects of negative pressure on junctional protein expression in primary human corneal epithelial cells.

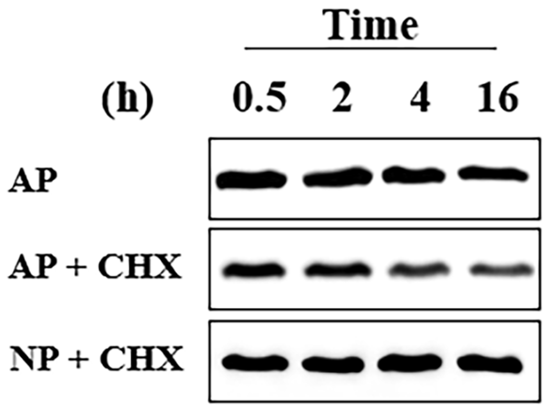

Figure S3. Representative western blot images of the cycloheximide chase assay showing G3BP1 protein stability under atmospheric pressure (AP) and negative pressure (NP) conditions.
